# Prediction of Temperature-Dependent Mechanical Properties for SWCNT/Cu Nanocomposite Metamaterials: A Molecular Dynamics Study

**DOI:** 10.3390/nano13121885

**Published:** 2023-06-19

**Authors:** Hai-Ning Zhang, Yin Fan, Hui-Shen Shen

**Affiliations:** School of Aeronautics and Astronautics, Shanghai Jiao Tong University, Shanghai 200240, China; ning-h-z@sjtu.edu.cn (H.-N.Z.); hsshen@sjtu.edu.cn (H.-S.S.)

**Keywords:** molecular dynamics simulation, chiral SWCNT reinforced nanocomposite, temperature-dependent mechanical properties, auxetic metamaterials

## Abstract

Single-walled carbon nanotube (SWCNT) is a promising candidate for strengthening nanocomposite. As the matrix of nanocomposite, a single crystal of copper is designed to be in-plane auxetic along the crystal orientation [1 1 0]. In that way, the nanocomposite could also be auxetic when enhanced by (7, 2) a single-walled carbon nanotube with relatively small in-plane Poisson’s ratio. A series of molecular dynamics (MD) models of the nanocomposite metamaterial are then established to study mechanical behaviors of the nanocomposite. In the modelling, the gap between copper and SWCNT is determined following the principle of crystal stability. The enhanced effect for different content and temperature in different directions is discussed in detail. This study provides a complete set of mechanical parameters of nanocomposite including thermal expansion coefficients (TECs) from 300 K to 800 K for five weight fractions, which is essential for a wide range of applications of auxetic nanocomposites in the future.

## 1. Introduction

Carbon nanotubes (CNTs) [1,2] have outstanding physical properties, such as superior mechanical behaviors, good electrical conductivity, and exceptional thermal performance, together with light weight [3]. Accordingly, for high-performance nanocomposites, CNTs are considered as ideal reinforcements to fulfill diverse requirements from high-end manufacturing [4]. Metal matrix nanocomposites (MMCs) reinforced by CNTs [5,6,7], which can serve in a high-temperature environment, have great potential in electronic, aerospace, and other industries. Experimental studies [8,9] have reported that only a small addition of CNT could result in a significant enhancement in mechanical properties of metals. Among MMCs, CNT reinforced copper (CNT/Cu) nanocomposites gain considerable attention due to their excellent electrical and thermal conductivity as well as high strength. Moreover, low TECs of CNT/Cu nanocomposites are favorable for reducing the strains caused by the TEC mismatch between electronic devices [10,11]. It is worth mentioning that the high performance of CNT/Cu nanocomposites is attributed to homogeneous distribution, improved interfacial reaction, and enhanced structural integrity of CNTs in copper matrix [12,13]. Therefore, many fabrication technologies have been developed to address these issues, such as molecular-level mixing [14,15,16,17], high-energy ball milling [18,19], electrochemical co-deposition [20], and spark plasma sintering [21]. Obviously, these experimental results are influenced by the processing method. In this case, an atomistic simulation like MD may be suitable for parametric investigations of the effects of composites and geometry on mechanical properties [22,23,24,25,26].

Most studies on mechanical behaviors of metal matrix nanocomposites, however, have not paid enough attention to a phenomenon of negative Poisson’s ratio (NPR). NPR, also known as “auxetic” [27], is an abnormal phenomenon where a material expands transversely under tension and contracts transversely under compression. NPR can be obtained through microstructural design, such as porosity and re-entrant, inside materials [28]. Hence, any material, theoretically, can be auxetic with a designed microstructure. Auxeticity can bring higher vibration absorption [29], increased indentation resistance [30], and improved plane strain fracture resistance [31]. However, there is a proverb that says you cannot have your cake and eat it too. Internal microstructure also means loss of material, inevitably resulting in degradation of stiffness and strength, but they are highly pursued in engineering. It is a contradiction that NPR and high stiffness and strength are mutually exclusive [32]. One solution is to adopt the sandwich structure that takes advantage of the unique properties of auxetic cores and composite face sheets [33]. Another solution is to design auxetic metamaterial in atomic scale to avoid porosity. In this way, the auxetic characteristics are obtained without sacrificing physical performance. For example, some metals with face-centered cubic (FCC) lattice have NPR along a non-axial direction [34,35]. Recently, based on MD simulations, Fan et al. [36,37] confirmed that single-crystal copper showed in-plane NPR of about −0.19 when subjected to uniaxial [1 1 0] loading.

It must be admitted that research on the mechanical behavior of CNT reinforced copper matrix composites has been relatively limited and the available data for further mechanical analysis are not comprehensive. In particular, the content of CNT plays a key role in strengthening the properties of composites. The main goal of this study is to design a SWCNT reinforced copper matrix (SWCNT/Cu) nanocomposite metamaterial and establish an effective corresponding molecular dynamics model to predict its mechanical properties, especially NPR, under different temperature. Additionally, the enhanced effect of SWCNT will be discussed in detail. A new design idea of auxetic materials is proposed to provide useful guidance for preparation.

## 2. Materials and Methods

**Figure 1:**
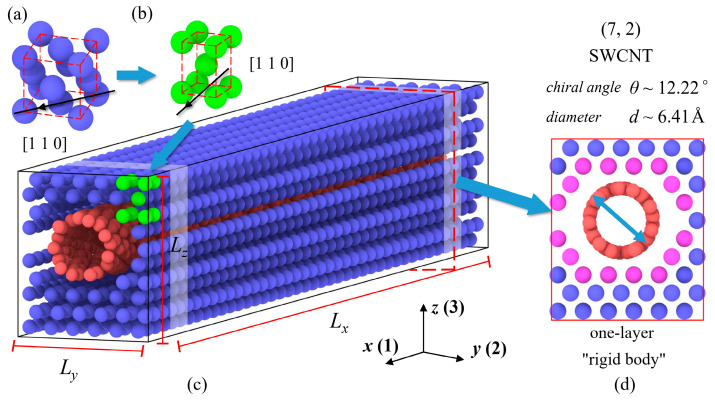
The schematic of the (**a**) single-crystal copper cell, (**b**) representative cell (green) along [1 1 0] crystal orientation in the SWCNT/Cu nanocomposite, (**c**) SWCNT/Cu nanocomposite (Cu atoms are in blue, and C atoms are in red), (**d**) front view of the nanocomposite (purple Cu atoms are set fixed after equilibration when simulating shear deformation).

In this study, an MD model of SWCNT/Cu nanocomposite is created along the *x*[1 1 0], *y*[1 $\bar{1}$ 0], and *z*[0 0 1] crystal orientations of single-crystal copper as depicted in Figure 1a. For simplicity, we utilize the numbers 1, 2, and 3 to represent the directions of *x*, *y*, and *z*, respectively. Thus, a new representative cell for copper matrix along the crystal orientations is shown in Figure 1b. To ensure that the nanocomposite has obvious auxetic properties, the Poisson’s ratio of SWCNT, according to rule of mixture, should be selected as small as possible. Furthermore, the minimum diameter for synthetic CNT is greater than 6 Å in the laboratory [38]. Therefore, the SWCNT with chiral indices (7, 2) is selected as a reinforcement in this article. The chiral angle *θ* and diameter *d* of (7, 2) SWCNT are 12.22° and 6.41 Å, respectively. According to our previous work [39], Poisson’s ratios for some chiral SWCNTs with variable chiral indices are listed in Table 1.

There are three potentials for atom pairs, i.e., Cu-Cu, C-C, and Cu-C, to be determined in this SWCNT/Cu nanocomposite model. The interaction between each pair of copper atoms is described by an embedded-atom method (EAM) potential [40,41], which is presented as:

(1) $$E_{\mathrm{Cu}}=F_{\alpha }\left[\sum_{j\neq i}\rho_{\beta }\left(r_{\;\mathrm{ij}}\right)\right]+\frac{1}{2}\sum_{j\neq i}\varphi_{\;\alpha \beta }(r_{\;\mathrm{ij}}),$$

where *E*_Cu_ is the total energy for a pair of Cu atoms, *F* is the embedding energy which is a function of the atomic electron density *ρ*, *φ* is a pair potential interaction between atoms *i* and *j* separated by a distance *r_ij_*, and subscripts *α* and *β* are the element types of atoms *i* and *j*. Conventionally, we use 3.615 Å and 63.546 as lattice constant and atomic weight of copper, respectively.

The adaptive intermolecular reactive empirical bond order (AIREBO) [42] potential with a cutoff radius (*r* = 3 Å) is adopted for the force field between carbon atoms, which is widely employed to study CNTs [22,23,24]. The cohesive energy of this potential consists of three terms:

(2) $$E_{\mathrm{CNTs}}=\frac{1}{2}\sum_{i}\sum_{j\neq i}\left[E_{\mathrm{ij}}^{\mathrm{REBO}}+E_{\mathrm{ij}}^{\mathrm{LJ}}+\sum_{k\neq i,j}\sum_{g\neq i,j,k}E_{\mathrm{kijg}}^{\mathrm{TORSION}}\right],$$

in which *E^REBO^*, *E^LJ^*, and *E^TORSION^* are potential energy from short-ranged reactive empirical bond order (REBO) potential, standard Lennard-Jones (LJ) potential that adds longer-ranged interactions and the torsional interactions term that describes various dihedral angle preferences in hydrocarbon configurations. The *E^REBO^* term in the AIREBO potential gives the model its reactive capabilities and only describes short-ranged interactions whose cutoff radii are less than 2 Å. The *E^REBO^* term is given as:

(3) $$E_{\mathrm{ij}}^{\mathrm{REBO}}=f(r_{\mathrm{ij}})(V_{\mathrm{ij}}^{R}+B_{\mathrm{ij}}V_{\mathrm{ij}}^{A}),$$

where *B^ij^*, $V_{\mathrm{ij}}^{R}$, and $V_{\mathrm{ij}}^{A}$ are the bond-dependent parameters which weigh the bond order, repulsive and attractive pair energy terms. *f* (*r_ij_*) is the cut-off function of the distance *r_ij_* between atoms *i* and *j* [42,43]. The C-C bond length used is a commonly accepted value of 1.42 Å, and the atom weight of C is 12.011.

To estimate the non-bonded interaction between copper and carbon atoms, we employ the LJ potential which has adequately predicted the Cu-C interaction and has been extensively used in the existing literature [22,23,24,25] on the MD study of carbon/copper molecular structures. The LJ potential energy is a function of the distance *r_ij_* between two atoms:

(4) $$E_{\mathrm{ij}}^{\mathrm{LJ}}=4\varepsilon \left[{\left(\frac{\sigma }{r_{\mathrm{ij}}}\right)}^{12}-{\left(\frac{\sigma }{r_{\mathrm{ij}}}\right)}^{6}\right],$$

in which *ε* and *σ* are the potential well depth and equilibrium interatomic distance, respectively. It is apparent that these two parameters have important consequences in the MD results. Here, we assign *ε* = 0.01996 eV and *σ* = 3.225 Å, which are also adopted in studies [44,45,46].

In this study, simulations are carried out using a large-scale atomic/molecular massively parallel simulator (LAMMPS) [47] and results are visualized by the Open Visualization Tool (OVITO) [48]. Periodic boundary conditions are set in the three directions to consider Poisson’s effect of material. Generally, there are two ways to present content of CNT in nanocomposites: volume fraction (vol.%) and weight fraction (wt.%). However, it might be inaccurate to measure SWCNT content by using volume fraction because determination of effective wall thickness of SWCNT is inevitable in this method but controversy truly exists [39]. Alternatively, the weight fraction of SWCNTs is adopted, which is obtained directly from the calculation based on the number of Cu and C atoms and their atomic mass. It should be noted that the weight fraction of CNT has an upper limitation to ensure stability of the whole system. Moreover, when designing MD models for SWCNT/Cu nanocomposites shown in Figure 1c, we use the common multiple of Cu lattice parameters and length of (7, 2) SWCNT to reduce the effect of mismatch in the longitudinal direction (i.e., *x*-axis) which may additionally cause the internal stress at the periodic interface. For instance, Figure 1c,d illustrates the three-dimensional structure and *yoz* section of the SWCNT/Cu nanocomposite with 8.29 wt.%. The simulation box containing 1681 Cu atoms and 804 C atoms is 104.8 × 15.3 × 18.1 Å^3^ in dimension. It should be pointed out that interface between Cu and SWCNT plays a key role in some mechanical properties, such as in-plane shear modulus. In this paper, determination of the gap will be discussed in detail in the next section. The temperature varying from 300 K to 800 K is also taken into account to evaluate the thermal effect on mechanical properties of nanocomposites. The duration of each timestep is set at 1 fs, and temperature and pressure of the system are both controlled by employing the Nosé–Hoover thermostat. The minimized structure is then equilibrated for a period of 5 × 10^4^ timesteps within the context of an isothermal-isobaric (NPT) ensemble.

After equilibration, the tensile and shear behaviors controlled by strain are applied to MD models to study in-plane and out-of-plane mechanical properties, containing Young’s modulus *E*, shear modulus *G*, and Poisson’s ratio *υ*. For tensile simulation, the specified constant engineering strain rate is 10^−5^/ps, and a 1% stretching strain is performed after 10^6^ timesteps in the NPT ensemble. Then, we can obtain Young’s moduli and Poisson’s ratios as:

(5) $$E_{\mathrm{ii}}=\frac{\sigma_{i}}{\varepsilon_{i}},$$

(6) $$\upsilon_{\mathrm{ij}}=-\frac{\varepsilon_{j}}{\varepsilon_{i}},$$

where subscripts *i* and *j* refer to 1, 2, and 3 directions (i.e., *x*, *y*, and *z*). Hence, there are three Young’s moduli and six Poisson’s ratios that need to be determined.

For shear testing, we also control the engineering strain rate to achieve the 1% shear strain. The calculation method of three shear moduli (*G*_12_, *G*_13_, and *G*_23_) is similar to Equation (5). It is emphasized that we set two “one-layer” of Cu and SWCNT sections, as shown in Figure 1d, in each MD model as “rigid” to restrain sliding of SWCNT during shear deformation. The sliding phenomenon means that only the interaction between Cu and C atoms works in resistance to shear deformation. In other words, the MD results only reflect the strength of the SWCNT/Cu interface without any limitations. On the other hand, the “one-layer” of “rigid” part should be as thin as possible to avoid unnecessary disturbance. Therefore, we utilize two “rigid” parts of thickness measuring 1.8 Å in the longitudinal direction (half of the copper lattice structure). Each part consists of 16 Cu atoms (marked as purple in Figure 1d). Furthermore, the thermal expansion coefficient (TEC) *α* and density *ρ* of nanocomposites can be obtained from the equilibration process as:

(7) $$\alpha_{\mathrm{ii}}=\frac{1}{L_{i0}}\frac{dL_{i}}{dT},$$

(8) $$\rho =\frac{n_{C}\;\cdot M_{C}+n_{\mathrm{Cu}}\;\cdot M_{\mathrm{Cu}}}{V\;\cdot N_{A}},$$

in which the unit of *α* and *ρ* are K^−1^ and g/cm^3^. *i* represents the 1, 2, and 3 directions, respectively. *n*, *L_i_*, and *V*, respectively, denote number of atoms, length in the *i* direction and volume of SWCNT/Cu nanocomposites. Correspondingly, *L_i_*_0_ represents the initial length of SWCNT/Cu nanocomposites. *T* is temperature with the unit of Kelvin and *N*_A_ is the Avogadro constant.

According to the density formula of nanocomposites (Equation (8)), the relationship between vol.% and wt.% of CNTs can be derived as:

(9) $$\mathrm{vol}.\%=\frac{\mathrm{wt}.\%}{\mathrm{wt}.\%+\left(\frac{\rho_{CNT}}{\rho_{Cu}}\right)-\left(\frac{\rho_{CNT}}{\rho_{Cu}}\right)\cdot \;\mathrm{wt}.\%}.$$

where *ρ_CNT_* and *ρ_Cu_* stand for the density of CNTs and Cu in nanocomposites. Generally, *ρ_CNT_* takes a value of 1.8–2.1 g/cm^3^ and *ρ_Cu_* is chosen as 8.9–8.96 g/cm^3^ [14,49].

## 3. Results and Discussion

### 3.1. Determination of Gap

**Figure 2:**
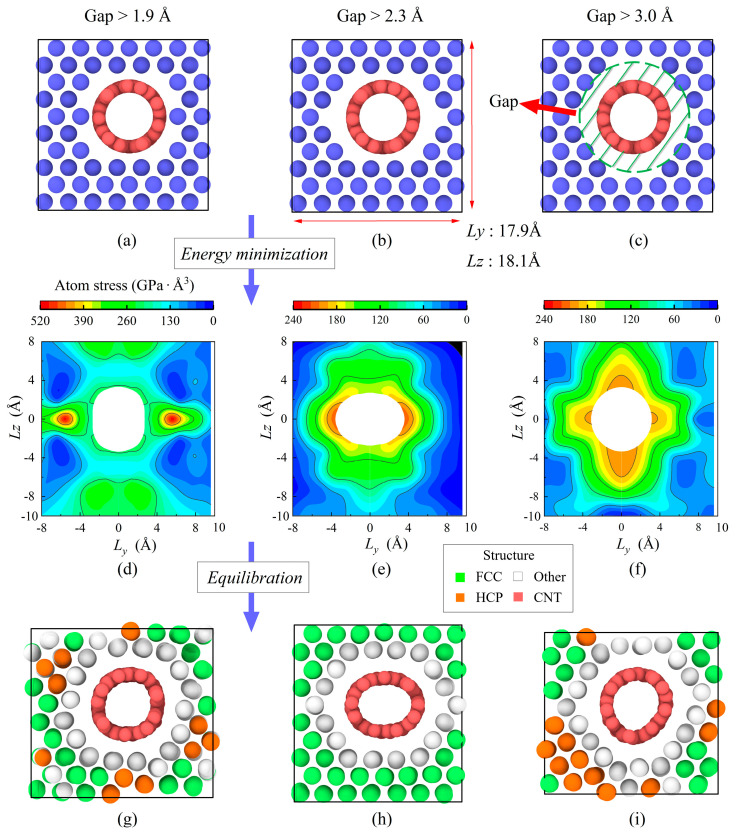
Different options of gap between Cu and SWCNT. (**a**–**c**) initial construction (green shade annulus represents gap area), (**d**–**f**) atomic stress distribution after energy minimization, and (**g**–**i**) crystal lattice structure after equilibration.

The interface between SWCNT and copper plays a fundamental role in some mechanical performance of nanocomposites [50], such as shear modulus. The selection criterion of minimum gap (distance) between copper atom and carbon atom is following the principle of a stable crystal system after relaxation. Three representative models with various gaps (i.e., 1.9 Å, 2.3 Å, and 3.0 Å) and their simulation results after relaxation at room temperature (300 K) are shown in Figure 2. They are the same size, 104.8 × 17.9 × 18.1 Å^3^, but have different weight fractions (6.5 wt.%, 6.8 wt.%, and 7.3 wt.%). Figure 2d–f shows the atomic stress distributions of the three models. In Figure 2d, the highest atom stress in the atomic structure with 1.9 Å gap is up to 520 GPa⸱Å^3^ after energy minimization. In contrast, models for 2.3 Å and 3.0 Å gaps have the distribution of atom stress at a lower level. Furthermore, although the cross-section of SWCNT in Figure 2f is well maintained in a circular shape, the nanocomposite with 3.0 Å gap does not have symmetric stress distribution in the *z*-direction. After equilibration, the structures with 1.9 Å and 3.0 Å gap both exhibit lattice deformation, shown in Figure 2g,i. One reason may be the performance difference due to the different lattice constants of [1 1 0] and [0 0 1] Cu crystal orientations. Therefore, 2.3 Å gap seems the best choice among the three from the view of system stability.

### 3.2. Effect of SWCNT Content

**Figure 3:**
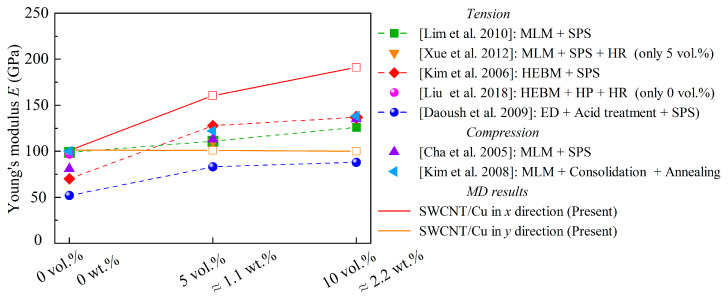
Comparison of Young’s modulus of CNT/Cu composites with varied CNT weight fractions at 300 K. MLM: molecular-level mixing; HEBM: high-energy ball milling; SPS: spark plasma sintering; ED: electroless deposition; HP: hot pressing; HR: hot rolling [14,15,16,17,18,19,21].

The correspondence between the present work and experiment results [14,15,16,17,18,19,21] is shown in Figure 3, where Young’s modulus of CNT/Cu composites synthesized by experiments are compared. In the figure, volume fractions of CNTs are converted to weight fractions by Equation (9) (i.e., 5 vol.% ≈ 1.1 wt.%; 10 vol.% ≈ 2.2 wt.%) for comparison. Although a difference does exist for various material systems, the Young’s moduli from the present work are close to those from mainstream experimental methods [14,15,16,17,18,19,21]. The figure also verifies the effectiveness of the present MD model.

**Figure 4:**
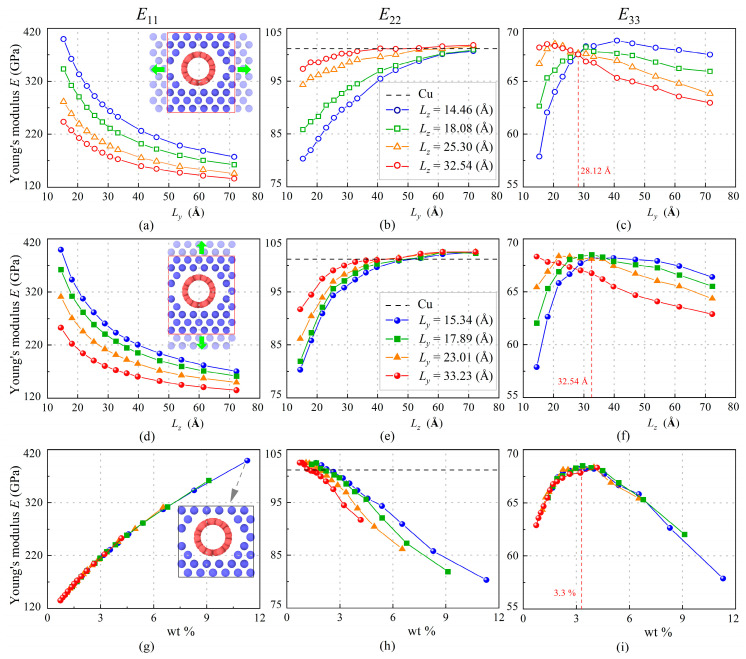
The variation of Young’s moduli *E*_11_ (**a**,**d**,**g**), *E*_22_ (**b**,**e**,**h**), and *E*_33_ (**c**,**f**,**i**) of SWCNT/Cu nanocomposites at 300 K with different lengths in *y*- and *z*-direction and corresponding SWCNT weight fractions (The green arrows denote the direction of size change).

To study the effect of SWCNT weight fraction on mechanical properties of SWCNT/Cu nanocomposite, 100 MD models are built and divided into four groups according to their lengths in the *y-* and *z*-directions (*L_y_* and *L_z_*), respectively. All the models are simulated at room temperature. Figure 4a,d,g show that Young’s modulus *E*_11_ drops when length (*L_y_* or *L_z_*) increases, which means the SWCNT weight fraction decreases simultaneously. The nanocomposite with 11.3 wt.% obtains a maximum *E*_11_ at about 399.3 GPa. For the group where *L_z_* = 14.46 Å in Figure 4a, *E*_11_ changes from 399.3 GPa to 260.5 GPa within the interval of 20 Å, which dropped by 35%. In the next interval, *E*_11_ reduces to 203.9 GPa, which dropped by 22%. The rate of decline slows slightly in the interval 50~70 Å. This trend is similar to the effect of *L_z_* in Figure 4d. In other words, the influence of size increase in any direction on *E*_11_ is similar. In contrast, *E*_22_ is improved as lengths (*L_y_* and *L_z_*) enlarge or wt.% decreases. It is apparent, in Figure 4e, that the sensitivity of *E*_22_ to lengths diminishes when lengths exceed 45 Å and the role of SWCNT turns from weakened to enhanced. After this, *E*_22_ remains at around 102.5 GPa, a little higher than copper (101.1 GPa). Unlike *E*_11_ and *E*_22_, both having a monotone relation to weight fraction, there is a maximum value of out-of-plane modulus *E*_33_ existing with the change of weight fraction. This trend was also observed in some experimental studies [16,21,49]. The composites with higher wt.% have lower relative density, which can be attributed to the original clustering of CNTs [51]. The maximum value is about 68.5 GPa at 3.3% weight fraction which is independent on *L_y_*. Thus, it is clear that the enhanced effect of the SWCNT to the auxetic copper is different in the three directions.

**Figure 5:**
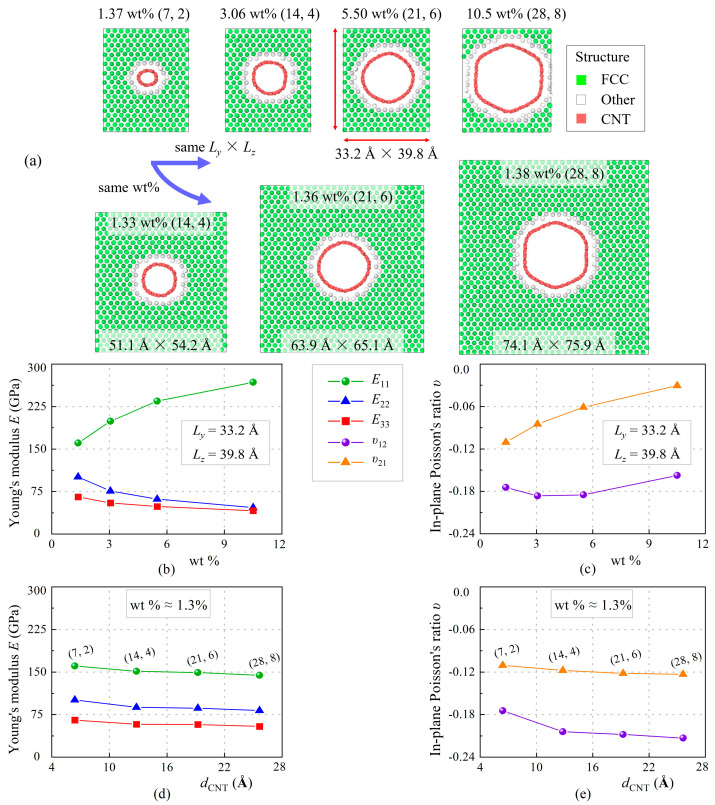
The variation of Young’s moduli and Poisson’s ratios of SWCNT/Cu nanocomposites at 300 K with different diameter and weight fraction of SWCNTs. (**a**) MD models for the nanocomposites with same size but different SWCNT or similar weight fraction but different size; (**b**) Young’s moduli and (**c**) in-plane Poisson’s ratios of the nanocomposites corresponding to the 4 models on the top in (**a**); (**d**) Young’s moduli and (**e**) in-plane Poisson’s ratios of the nanocomposites corresponding to the first model on the top left and 3 models on the bottom in (**a**).

There are two cases displayed in Figure 5a to study the effect of SWCNT size: (1) nanocomposites with same length but owning SWCNTs of larger diameter; (2) nanocomposites with same weight fraction but owning SWCNTs of larger diameter. In can be seen in Figure 5c that *E*_11_ enlarges with increasing wt.%, while *E*_22_ and *E*_33_ decline. However, *E*_11_ of nanocomposite with 10.5 wt.% (28, 8) SWCNT is only 267.8 GPa, about 60% *E*_11_ of nanocomposite with 11.3 wt.% (7, 2). The reason is that SWCNTs of small diameter possess higher modulus [39] and thus greater enhancement effects. As for the case of nanocomposites with the same weight fraction, all three moduli have been slightly reduced. In the meantime, negative Poisson’s ratios have not been well improved, considering the much larger size of the copper. Moreover, it can be observed that the larger the diameter, the more irregular the cross-section of SWCNT becomes after equilibration. This means that the overall structure is more prone to failure and therefore exhibit low strength. In conclusion, smaller diameter carbon tubes have greater enhancement potential [52].

### 3.3. Effect of Temperature

**Figure 6:**
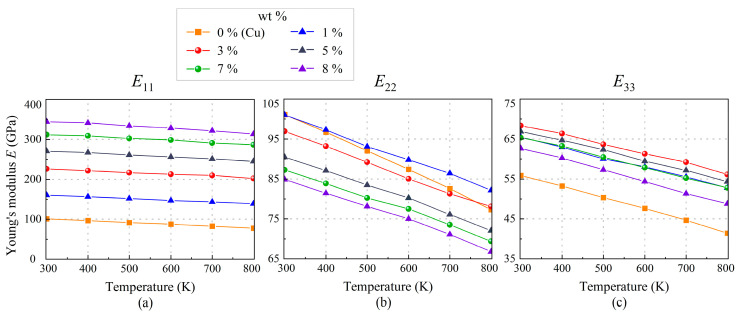
The variation of Young’s moduli: (**a**) *E*_11_, (**b**) *E*_22_, and (**c**) *E*_33_ of SWCNT/Cu nanocomposites with different SWCNT weight fractions under a thermal environment.

We simulate single-crystal copper and SWCNT/Cu nanocomposites with five SWCNT weight fractions of 1.37%, 3.35%, 4.96%, 6.78%, and 8.29% from 300 K to 800 K to predict temperature-dependent mechanical properties. The thickness to width ratio (*L_z_*/*L_y_*) is limited between 0.8 and 1.2 to eliminate size effect. The MD results of in-plane moduli are compared in Table 2 and plotted in Figure 6. It can be seen that the modulus of nanocomposites in the *x*-direction is significantly higher than that of pure copper. As expected, SWCNT/Cu nanocomposite with 8.29 wt.% has the largest *E*_11_ about 344.1 GPa, increased by 240% compared with pure copper (0 wt.%) at room temperature (300 K). When the temperature reaches 800 K, the enhanced effect achieves about 304%, which is consistent with the observation in [25]. Moreover, all three Young’s moduli decrease with the increase in temperature. It is easy to understand the weakened loading bearing capacity of SWCNT/Cu nanocomposites caused by the expansion of distances of atoms and softened crystalline structure due to raised temperature [24]. It is easy to find that the enhanced effect in the *x* direction is more remarkable than those in the other two directions from Table 2, that is because of the mechanical characteristics of the reinforcement SWCNT. In other words, contribution of SWCNT to *E*_11_ of nanocomposite is more than that of Cu. In addition, SWCNT contribution proportion is improved with higher content. For the cases of *E*_22_ and *E*_33_, however, contributions of Cu t are dominant. Furthermore, from Table 2, the degradation of mechanical property is sensitive to temperature for copper. However, it is reported that Young’s modulus of SWCNT in the longitudinal direction is only reduced by 3% when temperature is raised from 300 K to 700 K [53], which means longitudinal modulus of SWCNT is almost temperature independent. Hence, this explains well why Young’s modulus of SWCNT/Cu is almost temperature-independent in the *x*-direction but temperature-dependent in other directions.

**Figure 7:**
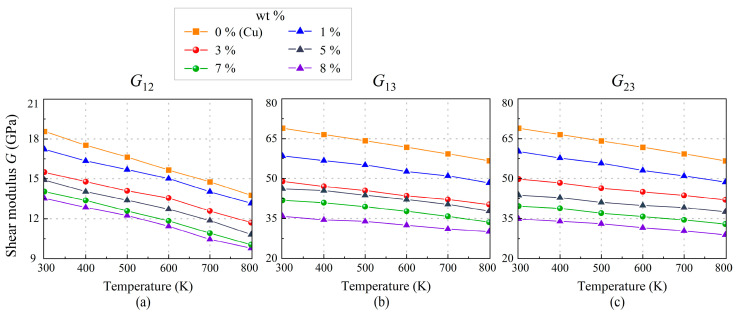
The variation of shear moduli: (**a**) *G*_12_, (**b**) *G*_13_, and (**c**) *G*_23_ of SWCNT/Cu nanocomposites with different SWCNT weight fractions under a thermal environment.

Due to the symmetrical lattice structure, *E*_11_ of copper is approximately equal to *E*_22_. However, we observe that the enhanced properties of SWCNT/Cu nanocomposites in the *x*-direction comes at the cost of a decrease in two/three-directional performance. For example, in the case of 8.29 wt.% SWCNT, *E*_11_ is almost four times that of *E*_22_ at room temperature. It is believed that SWCNT is a typical one-dimensional single-crystal nanomaterial and had greater excellent mechanical performance in the longitudinal direction. Figure 7 illustrates the effect of temperature and weight fraction on in-plane shear modulus *G*_12_ and out-of-plane shear moduli (*G*_13_ and *G*_23_). It was found that *G*_12_ is weakened by adding SWCNT reinforcement. This is because without intensive treatment the strength of the copper–carbon interface is very low due to poor wettability and interaction [12], which is precisely the decisive factor in resisting shear deformation. Therefore, the high strength of CNTs is not fully exploited [54]. *G*_12_ of 8.29 wt.% nanocomposite decreases from 13.5 GPa to 9.8 GPa when temperature increases to 800 K. Both in-plane and out-of-plane moduli of copper and SWCNT/Cu nanocomposites are provided in Table 2. It can be inferred that the performances of SWCNT/Cu nanocomposites and copper are close in the thickness direction while out-of-plane shear moduli of SWCNT/Cu nanocomposites are all lower than copper.

To compare the effect of SWCNT and defect (hole) on in-plane properties, five cases listed in Table 3 are investigated. The MD results in Table 3 reveal that the defect can significantly decrease the in-plane moduli and Poisson’s ratios of copper, which is in agreement with findings Zhao et al. [55] reported. It is apparent that there is no enhancement on *E*_22_ and *G*_12_ of nanocomposite with SWCNT. Inversely, these properties are degraded somehow compared with pure copper (Case 1). In other words, the effect of SWCNT in certain directions is similar to that of defect, although is not as obvious as that.

**Figure 8:**
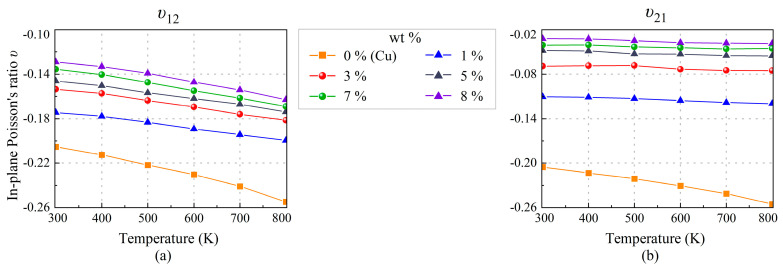
Thermal effect on in-plane Poisson’s ratios: (**a**) *υ*_12_ and (**b**) *υ*_21_ of nanocomposites with different SWCNT weight fractions.

In order to study the auxetic behavior of the SWCNT/Cu nanocomposites, in-plane Poisson’s ratios (*υ*_12_ and *υ*_21_) are depicted in Figure 8 and presented in Table 4. Single crystal copper, as a typical FCC metal, exhibits an in-plane auxetic behavior when the load is applied in the [1 1 0] direction. It is apparent that auxeticity is more significant with the increase in temperature. This phenomenon is also observed from the SWCNT/Cu nanocomposites. Furthermore, we find that the increase in SWCNT content may diminish the auxetic nature of SWCNT/Cu nanocomposites, although the Poisson’s ratio of SWCNT is positive but relatively small [39]. As listed in Table 3, the presence of nano-hole leads to NPRs *υ*_12_ and *υ*_21_ decreases by −19.4% and −37.0%, respectively. SWCNTs slightly improve the NPR *υ*_12_ but further weaken NPR *υ*_21_. In general, the NPRs SWCNT/Cu of composites are lower than that of the copper. Table 4 also presents the results of out-of-plane Poisson’s ratios from 300 K to 800 K. It should be pointed out that the limit of Poisson’s ratio from −1.0 to 0.5, which follows from stability conditions for isotropic materials, does not apply [37]. In addition, results in Table 2 and Table 4 reflect the remarkable anisotropy for the SWCNT/Cu nanocomposites, which is caused by the addition of SWCNTs.

**Figure 9:**
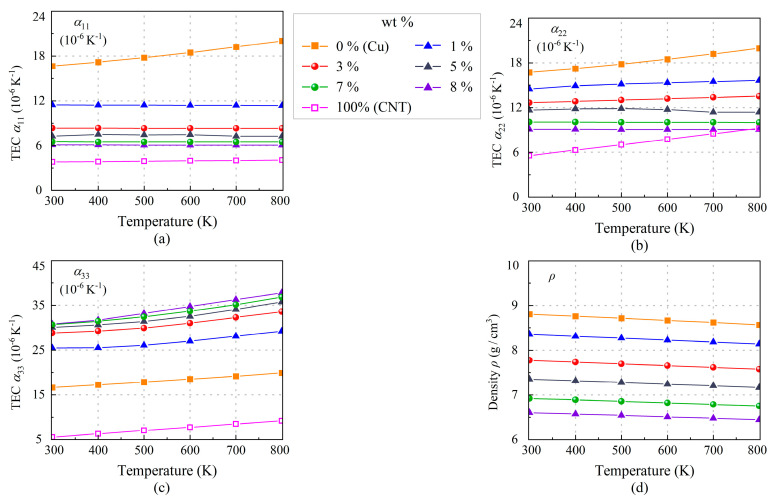
Thermal effect on TECs: (**a**) *α*_11_, (**b**) *α*_22_, (**c**) *α*_33_, and (**d**) *ρ* of SWCNT/Cu nanocomposites with different SWCNT weight fractions.

The TECs and density of (7, 2) SWCNT, copper, and the SWCNT/Cu nanocomposites varying from 300 K to 800 K are shown in Figure 9 and Table 5. Although TEC *α*_11_ of copper in Figure 9a is gradually enlarged as temperature rises, TEC *α*_11_ of SWCNT/Cu nanocomposites seems to be insensitive to temperature changes. It is evident that α_11_ is more sensitive to the SWCNT content, and for 8.29 wt.% at room temperature, *α*_11_ is just one third of that of copper. We find that *α*_22_ in Figure 9b is also insensitive to temperature changes but is larger than *α*_11_. In contrast, *α*_33_ shows a clear increasing trend as the temperature rises.

**Figure 10:**
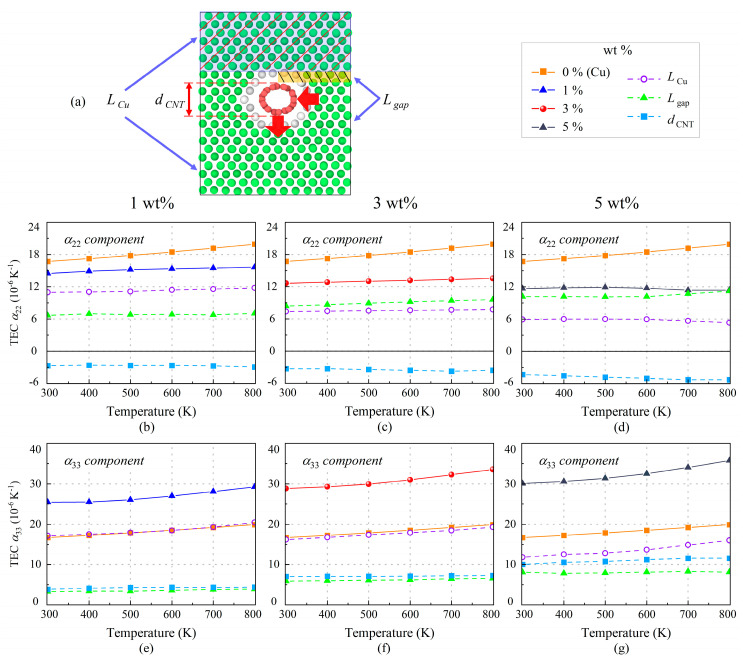
(**a**) Schematic graphic of three components for TECs of SWCNT/Cu nanocomposites. The effect of three components on TECs (**b**–**d**) *α*_22_ and (**e**–**g**) *α*_33_ of nanocomposites with 1 wt.%, 3 wt.%, and 5 wt.% SWCNTs.

In order to further analyze the structural changes of SWCNT/Cu nanocomposites in the temperature field, the model has been divided into three components as depicted in Figure 10a: the copper part (*L_Cu_*), the embedded SWCNT (*d_CNT_*), and the gap between copper and SWCNT (*L_gap_*). In fact, the sum of these three is the thickness *L_z_* of the model and the same is true in the *y*-direction. The detailed *α* of three components evaluated by Equation (7) under various temperatures are plotted in Figure 10. It is clear that the Cu part accounts for most of the variation in *α*_22_ and *α*_33_. However, it is gradually weakened with increased weight fractions. Correspondingly, the effect of the gap between copper and SWCNT becomes progressively greater, even over the Cu part results. The larger distance between the copper and SWCNTs leads to a weaker interface, which accounts for the diminished capacity of mechanical loading transfer for SWCNT/Cu nanocomposites. It is worth noting that *α*_22_ caused by SWCNT is negative, while *α*_33_ caused by SWCNT is positive, which means that the SWCNT expands in the *z*-direction but contracts in the *y*-direction.

## 4. Conclusions

An MD study on mechanical properties and TECs of a chiral SWCNT reinforced auxetic copper nanocomposite was carried out in this paper. In the modelling, an SWCNT with indices (7, 2) and FCC crystals of copper which have auxeticity in the [1 1 0] crystallographic orientation, were selected as the reinforcement and the matrix, respectively. The main conclusions can be drawn as follows:

1. To sustain the stability in subsequent mechanical behavior, it is most important to determine the gap between Cu and SWCNT according to minimization of energy. The comparison between three gaps on stress distribution and crystal deformation after relaxation shows that the gap of 2.3 Å is optimum in the present case. The enhanced effect of SWCNT on the considered nanocomposite in various direction is different.
2. As expected, elastic modulus *E*_11_ is significantly raised as it is along the length direction of SWCNT. In our simulation, *E*_11_ can reach about 399.3 GPa with 11.3 wt.% SWCNT, which is about four times that of the copper matrix. Whereas, *E*_22_ of nanocomposite is decreased because the effect of SWCNT in the *y*-direction is similar to a defect in this case. Unlike *E*_11_ and *E*_22_, *E*_33_ has an “inverse” trend with increase in SWCNT weight fraction, and the peak value of *E*_33_ is at 3.3 wt.% of around 68.5 GPa. These indicate that the considered nanocomposite is anisotropic in mechanics.
3. In addition, we discovered that smaller diameter CNTs have a better enhanced effect and its nanocomposite has more remarkable auxeticity. Although the SWCNT with positive Poisson’s ratio weakens the auxeticity of copper, *υ*_12_ of SWCNT/Cu can still reach about −0.2.
4. Finally, thermal effect is considered as temperature rises from 300 K to 800 K. With temperature increase, all elastic moduli and shear moduli are degraded due to the weakening of their own chemical bonds and interactions between SWCNT and Cu. However, at the same time, it is noted that the enhanced effect of SWCNT is relatively more obvious at a high temperature owing to the property degradation of SWCNT being less. On the whole, TECs *α*_11_ and *α*_22_ of nanocomposite are in between those of SWCNT and copper. However, an abnormal phenomenon is that TEC *α*_33_ is higher than that of any component material. Thus, we discussed the contributions of the three components (SWCNT, copper, and the gap) to the TEC in that direction to explain this phenomenon.

The MD model and material data of the nanocomposite metamaterial proposed in this paper can be referenced for further mechanical analysis.

## Figures and Tables

**Table 1:** Poisson’s ratios for chiral SWCNTs at 4.3 K [39] and 300 K (sorted by *υ*_12_).

Chiral Angle *θ*	Diameter *d* (Å)	(*n*, *m*)	*υ*_12_ at 4.3 K	*υ*_12_ at 300 K
10.89°	3.59	(4, 1)	0.0385	0.0437
8.95°	4.36	(5, 1)	0.0583	0.0699
19.11°	4.14	(4, 2)	0.0694	0.0792
7.59°	5.13	(6, 1)	0.0816	0.0952
16.10°	4.89	(5, 2)	0.0873	0.0988
6.59°	5.91	(7, 1)	0.1061	0.1106
13.90°	5.65	(6, 2)	0.1080	0.1143
5.82°	6.69	(8, 1)	0.1282	0.1301
12.22°	6.41	(7, 2)	0.1288	0.1312

**Table 2:** Temperature-dependent elastic moduli and shear moduli of SWCNT/Cu nanocomposites.

wt.%	*T* (K)	*E*_11_ (GPa)	*E*_22_ (GPa)	*E*_33_ (GPa)	*G*_12_ (GPa)	*G*_13_ (GPa)	*G*_23_ (GPa)
0%	300	101.10	101.14	55.890	18.562	68.975	68.946
	400	96.807	96.712	53.235	17.537	66.548	66.555
	500	91.809	92.039	50.318	16.635	64.198	64.150
	600	87.792	87.414	47.655	15.659	61.737	61.746
	700	82.777	82.590	44.673	14.762	59.318	59.303
	800	77.524	77.287	41.410	13.772	56.722	56.683
1%	300	160.47	101.02	65.478	17.218	58.517	60.179
	400	156.51	97.333	62.923	16.346	56.732	57.740
	500	151.69	93.078	60.041	15.691	55.113	55.789
	600	147.01	89.826	58.059	15.019	52.639	53.101
	700	143.65	86.397	55.533	14.037	51.043	51.025
	800	139.01	82.139	52.793	13.156	48.391	48.699
3%	300	226.24	96.918	68.336	15.496	48.932	49.838
	400	221.55	93.174	66.362	14.778	47.061	48.416
	500	216.70	89.186	63.629	14.090	45.508	46.357
	600	212.46	85.026	61.311	13.561	43.547	45.030
	700	209.84	81.311	59.185	12.584	42.145	43.688
	800	202.13	78.090	56.100	11.707	40.216	42.060
5%	300	270.58	90.433	66.897	14.914	46.189	43.764
	400	266.84	87.127	64.681	14.033	45.493	42.899
	500	261.25	83.460	62.333	13.397	43.732	41.059
	600	255.89	80.246	59.480	12.725	42.100	39.943
	700	251.02	76.117	57.167	11.876	40.322	39.127
	800	245.04	72.070	54.322	10.807	37.776	37.549
7%	300	312.01	87.278	65.314	14.039	41.793	39.616
	400	308.74	83.872	63.241	13.380	40.911	38.842
	500	302.61	80.254	60.448	12.593	39.407	37.000
	600	298.22	77.512	57.843	11.833	37.694	35.759
	700	290.73	73.516	55.218	10.909	35.857	34.479
	800	286.50	69.370	52.770	10.039	33.608	32.890
8%	300	344.09	84.881	62.647	13.531	35.872	34.853
	400	341.00	81.434	60.277	12.850	34.473	33.951
	500	334.00	78.165	57.300	12.243	33.924	33.109
	600	328.30	75.023	54.401	11.434	32.454	31.559
	700	321.57	71.129	51.350	10.443	31.072	30.365
	800	313.32	66.801	48.802	9.7921	30.050	28.922

**Table 3:** In-plane properties of copper, copper with the defect, and the SWCNT/Cu nanocomposite with 8.29 wt.% at 4.3 K.

Case	*E*_11_ (GPa)	*E*_22_ (GPa)	*G*_12_ (GPa)	*υ*_12_	*υ*_21_
Case 1	114.99	115.07	21.499	−0.1858	−0.1855
Case 2	84.649	53.750	2.9898	−0.1439	−0.0876
Case 3	361.13	67.517	10.969	−0.1506	−0.0287
Difference 2/1	−26.4%	−53.3%	−86.1%	−22.6%	−52.8%
Difference 3/2	326.6%	25.6%	266.9%	4.7%	−67.2%
Difference 3/1	214.1%	−41.3%	−49.0%	−18.9%	−84.5%
Case 4	95.885	74.560	8.4298	−0.1497	−0.1168
Case 5	360.21	92.907	16.061	−0.1200	−0.0316
Difference 4/1	−16.6%	−35.2%	−60.8%	−19.4%	−37.0%
Difference 5/4	275.7%	24.6%	90.5%	−19.8%	−72.9%
Difference 5/1	213.3%	−19.3%	−25.3%	−35.4%	−83.0%

Case 1: (104.80 × 15.34 × 18.08) Å^3^; 2460 Cu atoms. Case 2: (104.80 × 15.34 × 18.08) Å^3^; 1517 Cu atoms and 3.0 Å diameter hole. Case 3: (104.80 × 15.34 × 18.08) Å^3^; 1517 Cu atoms, 804 C atoms of SWCNT and 3.0 Å gap. Case 4: (104.80 × 15.34 × 18.08) Å^3^; 1681 Cu atoms and 2.3 Å diameter hole. Case 5: (104.80 × 15.34 × 18.08) Å^3^; 1681 Cu atoms, 804 C atoms of SWCNT and 2.3 Å gap. Difference *i*/*j* = [(*X_i_* − *X_j_*)/*X_j_*] × 100%; *X* = *E*, *G*, *υ*.

**Table 4:** Temperature-dependent Poisson’s ratios of SWCNT reinforced Cu nanocomposites.

wt.%	*T* (K)	*υ*_12_	*υ*_21_	*υ*_13_	*υ*_31_	*υ*_23_	*υ*_32_
0%	300	−0.2053	−0.2053	0.9216	0.4216	0.9215	0.4216
	400	−0.2125	−0.2133	0.9330	0.4228	0.9339	0.4232
	500	−0.2217	−0.2211	0.9476	0.4250	0.9467	0.4251
	600	−0.2304	−0.2308	0.9603	0.4262	0.9609	0.4265
	700	−0.2409	−0.2412	0.9763	0.4283	0.9766	0.4284
	800	−0.2547	−0.2553	0.9964	0.4309	0.9974	0.4310
1%	300	−0.1745	−0.1105	0.8326	0.2972	0.8370	0.4664
	400	−0.1779	−0.1113	0.8371	0.2971	0.8393	0.4672
	500	−0.1833	−0.1131	0.8470	0.2966	0.8460	0.4672
	600	−0.1894	−0.1156	0.8603	0.2941	0.8501	0.4721
	700	−0.1945	−0.1183	0.8664	0.2910	0.8527	0.4708
	800	−0.1996	−0.1198	0.8754	0.2899	0.8562	0.4744
3%	300	−0.1537	−0.0692	0.7508	0.2068	0.8121	0.4983
	400	−0.1574	−0.0688	0.7572	0.2043	0.8156	0.5017
	500	−0.1637	−0.0686	0.7677	0.2023	0.8215	0.5053
	600	−0.1695	−0.0734	0.7714	0.2006	0.8284	0.5073
	700	−0.1762	−0.0750	0.7833	0.2001	0.8316	0.5090
	800	−0.1814	−0.0752	0.7984	0.1980	0.8379	0.5102
5%	300	−0.1463	−0.0480	0.7075	0.1615	0.7975	0.5230
	400	−0.1505	−0.0487	0.7136	0.1599	0.8011	0.5233
	500	−0.1568	−0.0526	0.7243	0.1583	0.8066	0.5244
	600	−0.1620	−0.0529	0.7318	0.1565	0.8105	0.5279
	700	−0.1671	−0.0549	0.7457	0.1585	0.8187	0.5297
	800	−0.1739	−0.0554	0.7554	0.1588	0.8265	0.5300
7%	300	−0.1356	−0.0409	0.6667	0.1272	0.8064	0.5287
	400	−0.1405	−0.0405	0.6727	0.1278	0.8109	0.5312
	500	−0.1474	−0.0432	0.6810	0.1253	0.8148	0.5293
	600	−0.1551	−0.0446	0.6905	0.1209	0.8194	0.5321
	700	−0.1616	−0.0459	0.7097	0.1201	0.8253	0.5369
	800	−0.1691	−0.0455	0.7288	0.1224	0.8326	0.5361
8%	300	−0.1290	−0.0323	0.6324	0.1037	0.8189	0.5367
	400	−0.1332	−0.0326	0.6385	0.1043	0.8235	0.5381
	500	−0.1392	−0.0348	0.6479	0.1041	0.8301	0.5381
	600	−0.1472	−0.0377	0.6632	0.1023	0.8358	0.5393
	700	−0.1541	−0.0383	0.6795	0.1046	0.8388	0.5413
	800	−0.1629	−0.0388	0.7013	0.1059	0.8451	0.5411

**Table 5:** Temperature-dependent TECs of SWCNT/Cu nanocomposites.

wt.%	*T* (K)	*α*_11_ (×10^−6^ K^−1^)	*α*_22_ (×10^−6^ K^−1^)	*α*_33_ (×10^−6^ K^−1^)	*ρ* (g/cm^3^)
0%	300	16.659	16.715	16.677	8.8043
	400	17.183	17.229	17.245	8.7597
	500	17.783	17.812	17.847	8.7138
	600	18.459	18.462	18.486	8.6665
	700	19.206	19.174	19.165	8.6177
	800	20.024	19.944	19.886	8.5673
1%	300	11.433	14.483	25.461	8.3552
	400	11.420	14.899	25.504	8.3131
	500	11.407	15.165	26.073	8.2703
	600	11.394	15.338	26.996	8.2265
	700	11.381	15.476	28.104	8.1818
	800	11.368	15.636	29.228	8.1360
3%	300	8.3366	12.649	28.829	7.7709
	400	8.3296	12.825	29.266	7.7328
	500	8.3227	13.000	29.920	7.6940
	600	8.3158	13.175	31.003	7.6542
	700	8.3089	13.349	32.299	7.6134
	800	8.3020	13.522	33.597	7.5717
5%	300	7.2747	11.668	30.072	7.3495
	400	7.4919	11.823	30.590	7.3139
	500	7.4511	11.878	31.392	7.2778
	600	7.4719	11.742	32.544	7.2408
	700	7.2536	11.389	34.071	7.2030
	800	7.2484	11.376	35.754	7.1644
7%	300	6.5046	10.042	30.650	6.9240
	400	6.5004	10.031	31.444	6.8913
	500	6.4962	10.021	32.462	6.8580
	600	6.4920	10.011	33.698	6.8240
	700	6.4878	10.001	35.151	6.7893
	800	6.4835	9.9914	36.814	6.7539
8%	300	6.0873	9.084	30.815	6.6027
	400	6.0836	9.075	31.676	6.5722
	500	6.0799	9.067	33.193	6.5408
	600	6.0762	9.059	34.744	6.5087
	700	6.0725	9.051	36.278	6.4760
	800	6.0688	9.042	37.794	6.4430
100% (7, 2)	300	3.8150	5.5715 (*α*_22_ = *α*_33_)		−
	400	3.8641	6.2998 (*α*_22_ = *α*_33_)		−
	500	3.9131	7.0267 (*α*_22_ = *α*_33_)		−
	600	3.9621	7.7521 (*α*_22_ = *α*_33_)		−
	700	4.0110	8.4758 (*α*_22_ = *α*_33_)		−
	800	4.0599	9.1977 (*α*_22_ = *α*_33_)		−

## Data Availability

Any data included in the manuscript is adequately referenced and available.
